# Urinary androgens and breast cancer risk: results from a long-term prospective study based in Guernsey

**DOI:** 10.1054/bjoc.1999.1180

**Published:** 2000-04-03

**Authors:** D Y Wang, D S Allen, B L De Stavola, I S Fentiman, J Brussen, R D Bulbrook, B S Thomas, J L Hayward, M J Reed

**Affiliations:** Unit of Metabolic Medicine, St Mary's Hospital Medical School, London, W2 1PG, UK; Academic Oncology Unit, Guy's Hospital, Third Floor, Thomas Guy House, London, SE1 9RT, UK; Department of Epidemiology and Public Health, London School of Hygiene & Tropical Medicine, Keppel Street, London, WC1E 7HT, UK

**Keywords:** breast cancer risk, adrenal androgens, androsterone, aetiocholanolone, menopause

## Abstract

Between 1961 and 1967 a cohort of over 5000 women volunteered for a prospective study to determine the relationship between the urinary androgen metabolites, androsterone (A) and aetiocholanolone (E), and risk of breast cancer. During the first 10 years of the study the concentration of urinary A and E was determined in 1887 of the urine specimens. In 1971 we reported that subnormal amounts of urinary A and E were associated with a significantly increased risk of breast cancer. The cohort has been followed regularly during the 37 years since inception of the study and, by May 1998, 248 women had been diagnosed with breast cancer. Urinary androgen metabolites had been measured in 116 of these cases. Analysis of these data confirmed that women diagnosed in the first decade of the study were more likely to have low levels of urinary androgen metabolites. In the following decades, however, those who developed breast cancer were more likely to have manifested an increased A and E excretion. The reversal in the relationship between androgen metabolite excretion and risk suggests that age, or probably more importantly, menopausal status at diagnosis is an important modifying factor. Dichotomizing at age 50 it was found that in the younger age group (predominantly premenopausal) the rate ratios in the lowest tertile of A or E excretion were two- to threefold greater than for those in the highest tertile (χ^2^_1_= 3.57;*P* = 0.06: χ^2^_1_= 4.70;*P* = 0.03 for A and E respectively). In contrast, in the older age group comprising predominantly post-menopausal women, the rate ratios associated with the lowest tertile of A or E were half that of those in the highest tertile (χ^2^_1_= 4.10;*P* = 0.04; χ^2^_1_= 8.72;*P* = 0.003 for A and E respectively). This suggests that there may be different endocrine promotional factors for pre-and post-menopausal breast cancer. Hormonal risk factors may vary during the lifetime of an individual woman and this may have profound consequences for prevention strategies. © 2000 Cancer Research Campaign


British Journal of Cancer (2000) **82**(9), 1577–1584
© 2000 Cancer Research Campaign
DOI: 10.1054/ bjoc.1999.1180, available online at http://www.idealibrary.com on IDEAL®

### Urinary androgens and breast cancer risk: results from a long-term prospective study based in Guernsey

**DY Wang[1], DS Allen[2], BL De Stavola[3], IS Fentiman[3], J Brussen[3], RD Bulbrook[2], BS Thomas[2], JL Hayward[2] and MJ Reed[1]**

[1]Unit of Metabolic Medicine, St Mary's Hospital Medical School, London W2 1PG, UK; [2]Academic Oncology Unit, Third Floor, Thomas Guy House, Guy's Hospital, London SE1 9RT, UK; [3]Department of Epidemiology and Public Health, London School of Hygiene & Tropical Medicine, Keppel Street, London WC1E 7HT, UK

**Summary** Between 1961 and 1967 a cohort of over 5000 women volunteered for a prospective study to determine the relationship between the urinary androgen metabolites, androsterone (A) and aetiocholanolone (E), and risk of breast cancer. During the first 10 years of the study the concentration of urinary A and E was determined in 1887 of the urine specimens. In 1971 we reported that subnormal amounts of urinary A and E were associated with a significantly increased risk of breast cancer. The cohort has been followed regularly during the 37 years since inception of the study and, by May 1998, 248 women had been diagnosed with breast cancer. Urinary androgen metabolites had been measured in 116 of these cases. Analysis of these data confirmed that women diagnosed in the first decade of the study were more likely to have low levels of urinary androgen metabolites. In the following decades, however, those who developed breast cancer were more likely to have manifested an increased A and E excretion. The reversal in the relationship between androgen metabolite excretion and risk suggests that age, or probably more importantly, menopausal status at diagnosis is an important modifying factor. Dichotomizing at age 50 it was found that in the younger age group (predominantly premenopausal) the rate ratios in the lowest tertile of A or E excretion were two- to threefold greater than for those in the highest tertile ($\chi^2_1$ = 3.57; $P$ = 0.06: $\chi^2_1$ = 4.70; $P$ = 0.03 for A and E respectively). In contrast, in the older age group comprising predominantly post-menopausal women, the rate ratios associated with the lowest tertile of A or E were half that of those in the highest tertile ($\chi^2_1$ = 4.10; $P$ = 0.04; $\chi^2_1$ = 8.72; $P$ = 0.003 for A and E respectively). This suggests that there may be different endocrine promotional factors for pre-and post-menopausal breast cancer. Hormonal risk factors may vary during the lifetime of an individual woman and this may have profound consequences for prevention strategies. © 2000 Cancer Research Campaign

**Keywords:** breast cancer risk; adrenal androgens; androsterone;aetiocholanolone; menopause

It would be very useful if it were possible to identify, within an apparently normal population, those who will develop breast cancer. Although this can be achieved by genetic testing for mutations of BRCA1 and BRCA2, such susceptibility genes are involved in the aetiology of only about 5% of breast cancer cases (Lynch and Lynch, 1986). By contrast, events related to a woman's menstrual and reproductive history, which have been widely recognized as determinants of breast cancer incidence, are not impressive predictors of individual risks. Such poor predictive performance could result from these epidemiological variables being only manifestations of other underlying factors. Indeed the size of the effects of the menstrual and reproductive variables and the observation that breast cancer rates change over a woman's life have stimulated investigation of endogenous sex hormones as potential causes underlying these observed effects (Key and Pike, 1988; Toniolo et al, 1995; Key et al, 1996). Methodological issues related to hormone measurements, however, complicate the study of their relationship with breast cancer. Furthermore, there is no general consensus regarding which biological forms of these hormones are actually associated with the observed changes in incidence rates.

*Received 1 June 1999*
*Revised 6 December 1999*
*Accepted 17 December 1999*

*Correspondence to:* IS Fentiman

The role of steroids in the aetiology of breast cancer is uncertain. Extensive analysis of the scientific literature shows a highly significant increase in blood oestradiol levels in post-menopausal women with breast cancer (Thomas et al, 1997). The position of oestrogens and premenopausal women is uncertain (Key and Pike, 1988; Thomas et al, 1997). Androgens have been studied less extensively than oestrogens and no meta-analysis has yet been undertaken.

The first prospective study in this field was performed by Bulbrook and Hayward who showed that levels of androsterone (A) and aetiocholanolone (E) were abnormally low in women who subsequently developed breast cancer (Bulbrook and Hayward, 1967; Bulbrook et al, 1971). Most of the cases were premenopausal and the abnormality was present up to 11 years before the clinical diagnosis of the disease, suggesting that high levels of androgen metabolites might have a protective effect, at least in younger women. However, other mainly retrospective case–control studies have claimed that high androgen excretion is positively associated with breast cancer risk (e.g. Secreto and Zumoff, 1994). Such contrasting results could be reconciled if the effects of androgens depended on menopausal status. This would occur if the androgens acted as oestrogen inhibitors in premenopausal women and as oestrogen enhancers (or even oestrogens) in the post-menopausal (Secreto and Zumoff, 1994; Adams, 1998).

This paper is a further report on the prospective study of Bulbrook and Hayward involving the long-term follow-up of a

**Table 1** Follow-up time of the GI volunteers

| | All | | | Included only | | |
|---|---|---|---|---|---|---|
| | **Cases** | **Censored** | **Total** | **Cases** | **Censored** | **Total** |
| *n* | 248 | 4473 | 4921 | 116 | 1771 | 1887 |
| Follow-up (years) | | | | | | |
| Median | 23.4 | 33.4 | | 17.0 | 32.9 | |
| Interquartile range | (13.8–28.5) | (30.9–35.5) | | (9.4–26.5) | (31.2–35.6) | |

cohort of women living on the island of Guernsey and for whom urinary androgen metabolites are known. The cohort has been followed at regular intervals for the last 38 years and presents a unique opportunity to examine the long-term relationship between the excretion of urinary androgen metabolites and breast cancer risk.

### SUBJECTS AND METHODS

Between 1961 and 1967 over 5000 women from the Channel Island, Guernsey, volunteered to take part in a prospective study of hormones and risk of breast cancer. Participation in the study entailed collection of a 24-h urine specimen and self-completion of a semi-structured questionnaire, which included information about the woman's date of birth, height and weight (both self-reported), menstrual history (and number of days since last menses, if premenopausal), reproductive history, medical history (including family history of breast disease) and use of medication in the past year.

The urine specimens were frozen, then sent to the laboratories in London by air, where creatinine assays, to test for sample completeness, were performed. A small number of specimens were discarded on arrival in London due to thawing in transit, or because glucose or protein was present in the specimen. The final number of women held in the database for this Guernsey study (GI) is 4921.

### Follow-up

The cohort is being followed at 6-monthly intervals for diagnoses of cancer, vital status and causes of death. This involves obtaining pathology reports for cancer diagnoses from the island's only hospital, death certificates from the Director of Public Health for Guernsey, and registrations for Guernsey women from the Wessex Cancer Registry. Since 1996 all Guernsey cancers have been registered with the Wessex Cancer Registry, whereas previously only women having radiotherapy or other treatment in Southampton were so registered.

Changes of name through marriage or deeds poll were checked at the island registry, the Greffe. By going through the GP records from the six practices on the island it was estimated that follow-up was 96% complete (Wang et al, 1992). Due to lack of resources we have been unable to update this figure, but have no reason to believe it has changed appreciably. For the purposes of these analyses follow-up is to May 1998, with censoring at death or diagnosis of any cancer. The median follow-up time of the volunteers for whom there was no diagnosis of breast cancer is 33.4 years (interquartile range 30.9–35.5 years) (Table 1, 'All' columns).

### Assays

From 1962 to 1964 the androgens were measured by the method of Kellie and Wade (1957), using the fraction of urine containing the 17-oxosteroids, which had been stored at –20°C. From 1964 the assay of androgen metabolites was performed using the method of Thomas and Walton (1968). Comparison of these two methods showed that the correlation of results for androsterone (A), or aetiocholanolone (E), was highly significant ($r > 0.9$), and that the slopes were statistically indistinguishable from unity (Thomas and Bulbrook, 1966).

Because the earlier method was time-consuming, at the beginning of the study assays were performed as breast cancer cases were diagnosed ($n = 19$), together with appropriately selected matched controls (Bulbrook and Hayward, 1967). The method of Thomas and Walton (1968), together with an increase in funding and staffing levels made it possible to do assays in a systematic way between 1967 and 1971, starting from those urine samples collected in the early years but not yet analysed. A total of 2162 measurements of A and E were performed.

### Exclusions

Of the 4921 volunteers who participated in the study 409 were excluded, either because they were prevalent cases or because their underlying risk of breast cancer differed from that in the general population. The exclusions were applied as follows: 23 women were prevalent cases of cancer at entry into the study and a further three had breast cancer diagnosed within the first 6 months of follow-up and therefore were classified as prevalent cases *and excluded*; 364 women had had a hysterectomy and/or oophorectomy before entry into the study and 19 were of unknown menopausal status. Androgens were not measured on an additional 2625 specimens; 133 because their creatinine level was below 0.7 g per 24 h, four where creatinine was not measured, and 2490 due to lack of available funding for this purpose at that time.

Table 2 lists the main characteristics of the volunteers included in the analyses and of those who were excluded either because of ineligibility or lack of adequate androgen measurements. They are generally very similar. It should be noted, however, that 80% of the volunteers were premenopausal at entry to the study.

### Statistical methods

Examination of the frequency distributions of A and E revealed that they were skewed but that the distributions of their log-transformed values were symmetrical. Regression analyses of these log-transformed values therefore were used to evaluate their relationship with age and menopausal status and showed that these

 *© 2000 Cancer Research Campaign*

**Table 2** Characteristics of the GI volunteers (by exclusion)

| | Included | | Excluded | | | |
|---|---|---|---|---|---|---|
| | | | Ineligible | | No androgens[a] | |
| Subjects (*n* and (cases)) | 1887 | (116) | 409 | (12) | 2625 | (120) |
| Premenopausal (*n* and (%)) | 1514 | (80%) | – | – | 2150 | (82%) |
| Parous (*n* and (%)) | 1561 | (83%) | 317 | (78%) | 2122 | (81%) |
| Age at entry (Mean and (s.d.)) | 42.1 | (7.3) | 46.5 | (6.2) | 41.9 | (7.0) |
| Age at menarche (Mean and (s.d.)) | 13.3 | (1.5) | 13.3 | (1.6) | 13.4 | (1.5) |
| Age at first birth (Mean and (s.d.)) | 26.0 | (4.8) | 24.7 | (7.3) | 25.4 | (4.8) |
| Height (m) (Mean and (s.d.)) | 1.62 | (0.06) | 1.62 | (0.06) | 1.06 | (0.07) |
| Weight (kg) (Mean and (s.d.)) | 62.5 | (10.1) | 63.7 | (10.3) | 62.7 | (11.0) |
| BMI (kg $m^{-2}$) (Mean and (s.d.)) | 23.8 | (3.6) | 24.3 | (3.8) | 24.0 | (4.1) |

[a]Among those eligible.

variables needed to be accounted for when comparing androgen levels with respect to breast cancer risks. To achieve this we classified women according to strata defined by age and menopause categories and, within each stratum, assigned women into A and E tertiles, adapting the method described by Zhang et al (1997) (see Appendix). Stratum-specific rates for breast cancer incidence were then estimated for these age- and menopause-adjusted tertiles of androgen levels and for categories of the potential confounders for which information had been collected: age at menarche (≤ 12, 13, >13), parity (0, 1+), age at first birth (≤ 20, 21–25, > 25, nulliparous), self-reported height and weight (both in tertiles) and body mass index (BMI = (wt (kg))/(ht(m))$^2$; also in tertiles).

Cox regression models with current age as the time scale were then fitted to the data in order to estimate age-adjusted rate ratios (RRs) (Clayton and Mills, 1993). Because we excluded all cases that occurred within the first 6 months of follow-up, the age at entry into the study of all women included in the analyses was shifted forward by 6 months. Assessment of the Cox proportionality assumptions was performed by plotting separately the cumulative incidence curves of the A and E tertiles (Aalen, 1978). These revealed possible interactions between current age and the androgens, effects that were further investigated by analysing separately events that occurred before or after age 50 (see Appendix). Potential confounders were also included in the model. Assessment of linearity, heterogeneity and interaction were performed by likelihood ratio tests.

### RESULTS

### The effect of age and menopausal status on androgen levels

Both androgens generally decreased with age, reaching a plateau after the menopause. Figure 1 shows the log-transformed values of A plotted against the age at which the urine samples were collected and Figure 2 shows the same results for E. Two regression lines are shown for women who were pre- and post-menopausal at entry to the study. There was no significant change in A and E levels with age in post-menopausal women, although their overall level differed from that of premenopausal women. Exclusion of extreme values (i.e. lying outside the variable's 95% range) did not change the estimated intercepts or slopes. To examine the androgens' effects on breast cancer risk, while controlling for variations due to differences in age and menopausal status, we created age- and menopause-specific tertiles of the two androgens, as described in the Appendix.

### Risk of breast cancer and androgen levels

The relationship between the age and menopause-adjusted tertiles of A and E and breast cancer is shown in Table 3. It can be seen that large values of the androgens appear to increase risk of breast cancer, but these effects are moderate and not significant, without a significant linear trend ($\chi^2_1$ = 1.07, $P$ = 0.30, and $\chi^2_1$ = 3.07, $P$ = 0.08 respectively).

### Risk of breast cancer, androgen levels and the effect of confounding variables

The estimated RRs for the potential confounders revealed that late age at menarche and early age at first birth had significant protective effects (test for linear trend $\chi^2_1$ = 4.21, $P$ = 0.04, and $\chi^2_1$ = 15.80, $P$ < 0.001 respectively), with parity indicating a significant rate reduction of 40% (RR = 0.60, $P$ = 0.02, in line with most published results (Salber et al, 1969; Hsieh et al, 1990). The estimated effects of the self-reported anthropometric variables supported the evidence found in other studies of differential effects in pre- and post-menopausal women, despite being self-reported (Wang et al, 1997). BMI in particular showed significant protective effects for women who were premenopausal at entry (RR for the second and third tertiles of BMI versus the first tertile were: 0.79 (95% confidence interval (CI) 0.51–1.21), 0.47 (95% CI 0.27–0.82), test for linear trend $\chi^2_1$ = 7.37, $P$ = 0.01) and harmful

*© 2000 Cancer Research Campaign*

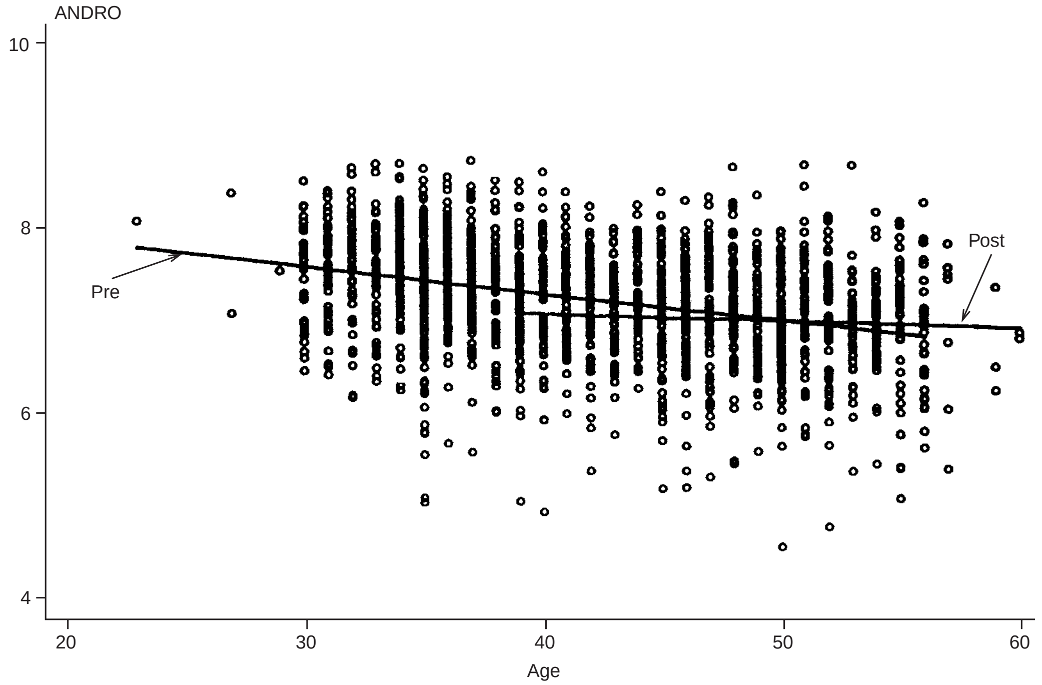

**Figure 1** Observed values of log-transformed ANDRO plotted against age and regression lines fitted on pre- and post-menopausal women ($n$ = 1514 and $n$ = 373 respectively). Estimated intercepts: 8.50, $P < 0.0001$, for pre-menopausal women, and 7.45, $P < 0.0001$, for post-menopausal women. Estimated slopes: –0.03, $P < 0.0001$, for pre-menopausal women, and –0.01, $P = 0.27$, for post-menopausal women

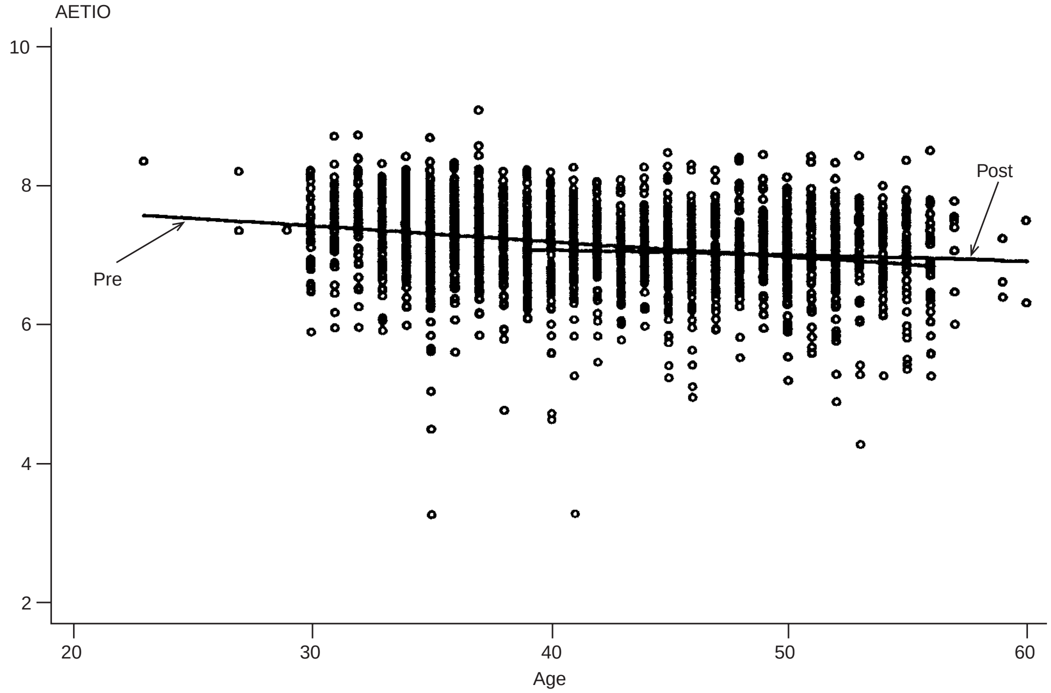

**Figure 2** Observed values of log-transformed AETIO plotted against age and regression lines fitted on pre- and post-menopausal women ($n$ = 1514 and $n$ = 373 respectively). Estimated intercepts: 8.11, $P < 0.0001$, for premenopausal women, and 7.41, $P < 0.0001$, for post-menopausal women. Estimated slopes: –0.03, $P < 0.0001$, for premenopausal women, and –0.01, $P = 0.32$, for post-menopausal women effects in those who were post-menopausal (RR for the second and third tertiles of BMI versus the first tertile were: 2.61 (95% CI 0.29–23.38), 6.35 (95% CI 0.81–49.63), test for linear trend $\chi^2_1 = 5.78$, $P = 0.02$). None of these factors, however, appeared to confound the estimated RRs for the androgens shown in Table 3.

 *© 2000 Cancer Research Campaign*

**Table 3** Distribution of breast cancer cases in the volunteers included in the analyses, associated rates and age-adjusted RRs by age and menopause specific tertiles of androgens

| Factor | Category | *n* | Cases | Rate (per 1000) | (95% CI) | RR | (95% CI) |
|---|---|---|---|---|---|---|---|
| Overall | | 1887 | 116 | 2.07[a] | (1.73–2.48) | | |
| ANDRO | 1st tertile | 635 | 35 | 1.88 | (1.35–2.61) | 1 | |
| | 2nd tertile | 644 | 38 | 1.98 | (1.44–2.72) | 1.05 | (0.66–1.66) |
| | 3rd tertile | 608 | 43 | 2.37 | (1.76–3.19) | 1.26 | (0.81–1.97) |
| Trend test | | | | | | $\chi^2_1 = 1.07$ | $P = 0.30$ |
| AETIO | 1st tertile | 635 | 33 | 1.77 | (1.26–2.49) | 1 | |
| | 2nd tertile | 645 | 36 | 1.85 | (1.34–2.57) | 1.04 | (0.65–1.68) |
| | 3rd tertile | 607 | 47 | 2.61 | (1.96–3.47) | 1.47 | (0.94–2.30) |
| Trend test | | | | | | $\chi^2_1 = 3.07$ | $P = 0.08$ |

[a]The overall crude rate may be moderately over-estimated due to the slight over-representation of early cases. However, the rate ratios should not be affected.

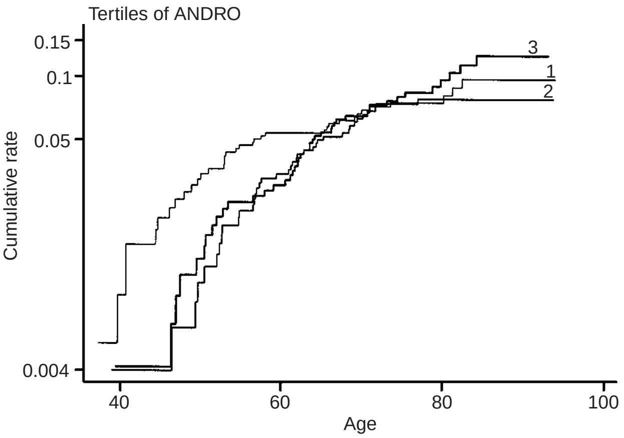

**Figure 3** Cumulative baseline rates of the three tertiles of ANDRO plotted on a log-scale. If the cumulative rates differed by a constant amount over the entire age period, the three curves should be parallel. Coding of ANDRO: 1 = 1st tertile, 2 = 2nd tertile, 3 = 3rd tertile

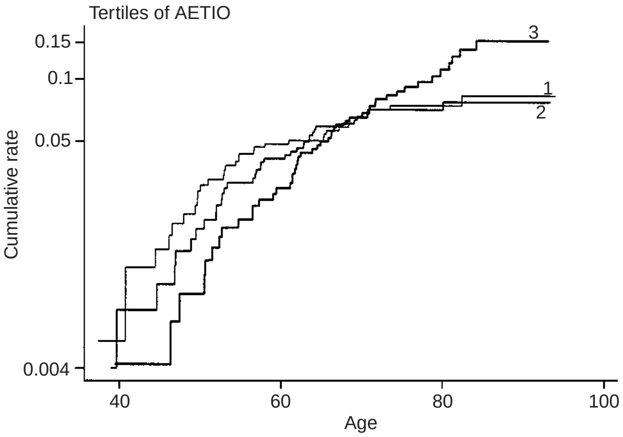

**Figure 4** Cumulative baseline rates of the three tertiles of AETIO plotted on a log-scale. If the cumulative rates differed by a constant amount over the entire age period, the three curves should be parallel. Coding of AETIO: 1 = 1st tertile, 2 = 2nd tertile, 3 = 3rd tertile

### Risk of breast cancer, androgen levels and the effect of current age

When the cumulative incidence rates for the separate tertiles of the two androgens were plotted (on a log scale) to assess the proportionality assumption underlying the Cox model, they converged and crossed, from around the time of menopause (Figures 3 and 4).

To assess this formally, separate Cox regression models were fitted after splitting breast cancer events and follow-up data according to whether they occurred before or after (and including) age 50, where 50 was chosen as the best estimate of the cohort average age at menopause. Table 4 shows the results of these age-band-specific estimates. In this analysis the RRs showed significantly protective effects of high androgen levels in younger women and significant harmful effects in older ones (linear tests for trend when current age < 50, $\chi^2_1 = 3.57$, $P = 0.06$, and $\chi^2_1 = 4.70$, $P = 0.03$ respectively; linear tests for trend when current age ≥50: $\chi^2_1 = 4.10$, $P = 0.04$, and $\chi^2_1 = 8.72$, $P = 0.003$ respectively). Note that 80 out of the 101 women were premenopausal at entry but had a diagnosis of breast cancer after age 50. They only moderately differed in terms of body size, but not of androgen tertiles nor age at first birth, from the cases who were already post-menopausal at entry. However, in order to interpret these results, the RRs were re-estimated using the subset of 382 women who were age 50 or older at entry. Among them, 19 had a diagnosis of breast cancer during their follow-up. The results were consistent with those shown in Table 4 for age ≤ 50 years, especially considering the smaller numbers involved (RRs for A: 0.96 (95% CI 0.31–2.91) and 1.25 (95% CI 0.42–3.72), test for linear trend, $P = 0.68$; RRs for E: 1.37 (95% CI 0.42–4.49) and 1.87 (95% CI 0.61–5.71), test for linear trend, $P = 0.27$). Tests for interaction between the two age-bands (before and after age 50) and the androgens were highly significant ($\chi^2_2 = 8.40$, $P = 0.015$, and $\chi^2_2 = 10.22$, $P = 0.006$ respectively). Furthermore, the results were not affected by the inclusion in the models of reproductive or anthropometric variables, so no confounding due to these factors could explain the results. When BMI was added to the two models of Table 4, the estimated effects of the two androgens were not substantially changed, indicating lack of confounding. Indeed, there was no clear association in our data between BMI and either of the two androgens, despite some evidence that the effect of BMI changed with age.

### DISCUSSION

The present results indicate a complex relationship between the level of urinary androgen metabolite excretion and risk of breast

© 2000 Cancer Research Campaign

**Table 4** Age-band specific rates and rate ratios (RRs) for age and menopause specific tertiles of androgens

| | Age-band < 50 | | | | | Age-band ≥ 50 | | | | |
|---|---|---|---|---|---|---|---|---|---|---|
| **Overall** | **Cases 21** | **Rate 1.40**[a] | **(95% CI) (0.91–2.15)** | **RR** | **(95% CI)** | **Cases 95** | **Rate 2.31**[a] | **(95% CI) (1.89,2.83)** | **RR** | **(95% CI)** |
| ANDRO (tertiles) | | | | | | | | | | |
| 1st | 12 | 2.43 | (1.38–4.27) | 1 | | 23 | 1.68 | (1.12–2.53) | 1 | |
| 2nd | 4 | 0.78 | (0.29–2.08) | 0.32 | (0.10–0.99) | 34 | 2.41 | (1.72–3.38) | 1.43 | (0.84–2.43) |
| 3rd | 5 | 1.02 | (0.43–2.45) | 0.42 | (0.15–1.19) | 38 | 2.86 | (2.08–3.94) | 1.70 | (1.02–2.86) |
| Trend test | | | | | $\chi^2_1 = 3.57$ ($P = 0.06$) | | | | | $\chi^2_1 = 4.10$ ($P = 0.04$) |
| Test for interaction | | | | | $\chi^2_2 = 8.40$ ($P = 0.015$) | | | | | |
| AETIO (tertiles) | | | | | | | | | | |
| 1st | 11 | 2.22 | (1.23–4.01) | 1 | | 22 | 1.61 | (1.06–2.45) | 1 | |
| 2nd | 7 | 1.36 | (0.65–2.85) | 0.61 | (0.24–1.57) | 29 | 2.03 | (1.41–2.93) | 1.26 | (0.73–2.20) |
| 3rd | 3 | 0.62 | (0.20–1.91) | 0.28 | (0.08–0.99) | 44 | 3.35 | (2.49–4.50) | 2.08 | (1.24–3.46) |
| Trend test | | | | | $\chi^2_1 = 4.70$ ($P = 0.03$) | | | | | $\chi^2_1 = 8.72$ ($P = 0.003$) |
| Test for interaction | | | | | $\chi^2_2 = 10.22$ ($P = 0.006$) | | | | | |

[a]The overall crude rate may be moderately over-estimated due to the slight over-representation of early cases. However, the rate ratios should not be affected.

cancer. In the first decade of follow-up, low levels of A and E are associated with an increase in the risk of disease while in the third decade this tendency is reversed. Why the predictive value of A and E should undergo such a reversal can only be speculated upon. It is unlikely to be methodological since all of the urinary assays were performed in the first decade of the follow-up period.

Implicit in these suggestions is the assumption that the level of A and E excretion (and androgen secretion) is characteristic of a woman; thus a high secretor will remain so, or relatively so, permanently. For many obvious reasons there are no experimental data on the changes in androgen production in a cohort of women for a period of time as long as 35 years. However, we do have indirect evidence that supports this assumption. A total of 294 women from the present cohort gave blood specimens for another phase of the research study about 15 years later. Concentrations of dehydroepiandrosterone (D) and dehydroepiandrosterone sulphate (DS) were measured on these samples. We have found a highly significant association between these blood androgens and the levels of urinary A and E in these women (Cramer's V (test of association) = 0.269, 0.278, 0.211, 0.236, all $P < 0.001$, for A with D, A with DS, E with D, E with DS respectively).

Over 80% of the cohort were premenopausal at entry into the trial which implies that women who developed breast cancer early in the follow-up period would have been predominantly premenopausal. As the follow-up time increased, the cohort would have passed through the menopausal years with the effect that later cases would have been post-menopausal at diagnosis. Thus a possible explanation could be that the relationship between androgen levels and breast cancer risk varies according to menopausal status.

The notion that there are two types of breast cancer, defined by menopausal status, has been in the scientific literature for over 30 years and recently reviewed by Adams (1998). The hypothesis was advanced that premenopausal breast cancer was the result of an endocrine dysfunction involving ovarian oestrogens, whereas the post-menopausal type involved adrenal oestrogens (De Waard et al, 1960, 1964). A two-disease mathematical model has been described by Manton and Stallard (1980) based on American breast cancer mortality rates which successfully predicted the age frequency of breast cancer deaths over an age range of 25–94 years.

In a review of the evidence for the existence of two types of human breast cancer, Adams (1983) compared the high breast cancer rates of Western Europe with the low rates in countries such as Japan. He points out that, post-menopausally, the European age-specific rates form approximate straight lines after subtracting the Japanese rates. These rates are termed 'Western environmental type' by De Waard (1969). This could imply that postmenopausal breast cancer might be the result of prolonged hormonal and environmental factors. This would be consistent with the report of Hayward et al (1978) that the lower incidence of breast cancer in post-menopausal Japanese women, living in Japan, compared to Western women, and the rise in incidence in migratory Japanese women is reflected in the levels of serum DS. It is possible, therefore, that low urinary androgen metabolites predict a high risk of early onset breast cancer, and that high levels are associated with an increased risk in the late, or post-menopausal, disease.

There are a large number of reports associating high androgen status in post-menopausal women with breast cancer (Grattarola et al, 1974; McFadyen et al, 1976; Adami et al, 1979; Secreto et al, 1983, 1991; Hill et al, 1985; Secreto and Zumoff, 1994; Dorgan et al, 1996; Thomas et al, 1997; Zeleniuch-Jacquotte et al, 1997). The post-menopausal type of disease could be influenced by oestrogens from the metabolism of adrenal androgens. This could come about by two pathways. The first is the aromatization of dehydroepiandrosterone by breast and adipose tissue and the conversion of oestrone to oestradiol. Thomas et al (1997) have reported a significant correlation between the serum levels of oestradiol and testosterone. The second mechanism derives from 5-androstene-3β,17β-diol, an important metabolite of dehydroepiandrosterone. The former steroid can compete with oestradiol for oestrogen receptors. Earlier studies demonstrated unique

 *© 2000 Cancer Research Campaign*

oestrogenic effects in the rat (Huggins et al, 1954), and more recently that, at concentrations found in the blood of Western women, it both increased uterine growth and DNA synthesis in this species (Seymour-Munn and Adams, 1983). Because of the androgenic origins of this oestrogen-like compound it has been dubbed 'hermaphrodiol' by Adams (1983). In normal breast tissue there is a highly significant correlation between the concentrations of D (or its sulphate ester) and 5-androstene-3β,17β-diol diol (Bonney et al, 1984). Thus high levels of D imply correspondingly increased concentrations of 5-androstene-3β,17β-diol. It has already been shown that there is a highly significant correlation between the amount of blood-borne D (or its sulphate ester) and the concentration of urinary A or E excreted (Wang et al, 1979). For this reason, the high urinary excretion of A or E could imply increased steroid substrates for oestradiol and 5-androstene-3β,17β-diol.

The initial results of the study (Bulbrook and Hayward, 1967; Bulbrook et al, 1971) came mainly from premenopausal women in whom a low discriminant function (i.e. low androgen relative to hydroxycorticoid excretion) was associated with increased breast cancer risk. The discriminant function reflects the relative secretions of D and cortisol. Reed (1995) has suggested that these steroids have a profound effect on T-helper cells which can express either a Th1 or a Th2 phenotype. Th1 and Th2 cells are characterized by the secretion of a unique battery of cytokines. Th1 cells secrete interferon γ (IFN-γ), interleukin-2 (IL-2) and tumour necrosis factor β (TNF-β) whilst Th2 cells produce IL-4, IL-5, IL-6 and IL-10. Th1 and Th2 cells tend to be mutually exclusive in that IFN-γ (Th1) inhibits the secretion of Th2 cytokines whilst IL-10 (Th2) inhibits the Th1 cytokine response. The paradigm suggested by Reed is that a low androgen to cortisol ratio would favour Th2 cells and the production of IL-6, a cytokine which up-regulates oestrogen synthesis in breast tissue.

The present results suggest that the endocrine components of the aetiology of early and late onset breast cancer may be different. If premenopausal breast cancer is the result of the effects of cytokines against the background of full ovarian activity and post-menopausal disease is the long-term effect of slight hyperoestrogenization then this could have a profound impact on prevention. It might explain why the incidence of genetically driven disease, predominantly premenopausal, is not significantly reduced by the administration of tamoxifen (Powles et al, 1998; Veronesi et al, 1998). In contrast to these negative findings, the NSABP P1 trial which included substantial numbers of post-menopausal women (one of the entry criteria being aged > 60), showed a significant reduction in incidence of breast cancer in all age groups given tamoxifen (Fisher et al, 1998). Our findings suggest that endocrine factors which are protective in premenopausal women may reverse their role after the menopause leading to the emergence of an increased risk of developing breast cancer.

### ACKNOWLEDGEMENTS

We thank the women of Guernsey who participated in this study together with Dr Bryan Gunton-Bunn and Dr David Jeffs for follow-up information. The authors are indebted to Professor J.B. Adams for his comments on the paper. This work was funded by both the Imperial Cancer Research Fund and Lloyds TSB Foundation for the Channel islands. DYW acknowledges financial support from the Breast Cancer Research Trust.

### APPENDIX

### Tertile analysis

The individual androgen levels, measured at entry to the study, were standardized as follows. We classified the women according to several strata; firstly by menopausal status (pre and post) and then, within each menopausal group, into several categories of age at entry (from age 30 to age 55 in 2-yearly intervals plus an additional category for age 56–60). Within each stratum we ranked the women according to their A values and classified them as belonging to either the first, second or third tertile. The same method was used for E. Hence, the observed values of both androgens were categorized into tertiles after accounting for the effect of age and menopausal status.

### Age-specific Cox regressions

To fit separate Cox regression models over the two separate age intervals, before 50 and after and including 50, we manipulated the data as follows:

- If a woman was younger than 50 years at entry to the study, and was still followed up after her 50th birthday, her follow-up information was split into two parts. The first would hold the length of the follow-up to age 50, with an indicator that she was disease-free at that age; the second part would indicate the length of the remaining follow-up time after age 50 and whether or not she was still disease-free at the end. Thus, if she had cancer diagnosed during the latter period, her event would appear in the analyses for age 50 or over
- If a woman was younger than 50 at entry, but her follow-up ended before her 50th birthday, no data expansion would be made. If she had been diagnosed with breast cancer at the end of her follow-up, her event would contribute to the number of pre-age 50 events.

If a woman was older than 50 at entry no data expansion was made. If she had been diagnosed with breast cancer at the end of her follow-up, her event would contribute to the number of post-age 50 events.

### REFERENCES

Aalen O (1978) Non-parametric inference for a family of counting processes. *Ann Stat* **6**: 701–726

Adami HO, Johansson ED, Vegelius J and Victor A (1979) Serum concentration of estrone, androstenedione, testosterone and sex-hormone-binding globulin in postmenopausal women with breast cancer in age-matched controls. *Upsala J Med Sci* **84**: 259–274

Adams JB (1983) Hermaphrodiol: a 'new' estrogen and its role in the etiology of human breast cancer. In: *Commentaries on Research in Breast Diseases* Bulbrook RD, Taylor DJ (eds), pp. 131–161. Alan R. Liss: New York

Adams JB (1998) Adrenal androgens and human breast cancer: a new appraisal. *Breast Cancer Res Treat* **51**: 183–188

Bonney RC, Scanlon MJ, Reed MJ, Jones DL, Beranek PA and James VHT (1984) Adrenal androgen concentrations in breast tumours and in normal breast tissue. The relationship to oestradiol metabolism. *J Steroid Biochem* **20**: 501–504

Bulbrook RD and Hayward JL (1967) Abnormal urinary steroid excretion and subsequent breast cancer. A prospective study in the Island of Guernsey. *Lancet* **i**: 519–522

Bulbrook RD, Hayward JL and Spicer CC (1971) Relation between urinary androgen and corticoid excretion and subsequent breast cancer. *Lancet* **ii**: 395–398

Clayton B and Mills M (1993) *Statistical Models in Epidemiology*. Oxford University Press: Oxford
De Waard F (1969) The epidemiology of breast cancer: review and prospects. *Int J Cancer* **40**: 577–586
De Waard F, deLaive JWJ and Baanders van Halewjin EA (1960) On bimodal age distribution of mammary carcinoma. *Br J Cancer* **14**: 437–448
De Waard F, Baanders van Halewjin EA and Huizinga J (1964) The bimodal age distribution of patients with mammary carcinoma. *Cancer* **17**: 141–151
Dorgan JF, Longcope C, Stephenson HE, Falk RT, Miller R and Sciajno R (1996) Relation of prediagnostic serum estrogen and androgen levels to breast cancer risk. *Cancer Epidemiol Biomarkers Prevent* **5**: 533–539
Fisher B, Costantino JP, Wickerham DL, Redmond CK, Kavanah M, Cronin WM, Vogel V, Robidoux A, Dimitrov N, Atkins J, Daly M, Wieand S, Tan-Chiu E, Ford L and Wolmark N (1998) Tamoxifen for prevention of breast cancer: report of the National Surgical Adjuvant Breast and Bowel Project P-1 study. *J Natl Cancer Inst* **90**: 13712–13788
Grattarola R, Secreto G, Recchione C and Castellini W (1974) Androgens in breast cancer II. Endometrial adenocarcinoma and breast cancer in married postmenopausal women. *Am J Obstet* **118**: 173–178
Hayward JL, Greenwood FC, Glober G, Stemmerman G, Bulbrook RD, Wang DY and Kumaoka S (1978) Endocrine status in normal British, Japanese and Hawaiian-Japanese women. *Eur J Cancer* **14**: 1221–1228
Hill P, Garbaczewski L and Kasumi F (1985) Plasma testosterone and breast cancer. *Eur J Cancer Clin Oncol* **21**: 1265–1266
Hsieh C-C, Trichopoulos D, Katsouyanni K and Yuasa S (1990) Age at menarche, age at menopause, height and obesity as risk factors for breast cancer: associations and interactions in an international case–control study. *Int J Cancer* **46**: 796–800
Huggins C, Jensen CV and Cleveland AS (1954) Chemical structure of steroids in relation to promotion of growth of the vagina and uterus of the hypophysectomized rat. *J Exp Med* **100**: 225–240
Kellie AE and Wade AP (1957) The analysis of urinary 17-oxosteroids by gradient elution. *Biochem J* **66**: 196–206
Key TJA and Pike MC (1988) The role of oestrogens and progestagens in the epidemiology and prevention of breast cancer. *Eur J Cancer Clin Oncol* **24**: 29–43
Key TJA, Wang DY, Brown JB, Allen DS, Moore JW, Bulbrook RD, Fentiman IS and Pike MC (1996) A prospective study of oestrogen excretion and breast cancer risk. *Br J Cancer* **73**: 1615–1619
Lynch HT and Lynch JF (1986) Breast cancer genetics in an oncology clinic: 328 consecutive patients. *Cancer Genet Cytogenet* **23**: 369–372
Manton KG and Stallard E (1980) A two-disease model of female breast cancer: mortality in 1969 among white females in the United States. *J Natl Cancer Inst* **64**: 9–16
McFadyen IJ, Forrest AP, Prescott RJ, Golder MP, Groom GV, Fahmy DR and Griffiths K (1976) Circulating hormone concentrations in women with breast cancer. *Lancet* **i**: 1100–1102
Powles T, Eeles R, Ashley S, Easton D, Chang J, Dowsett M, Tidy A, Viggers J and Davey J (1998) Interim analysis of the incidence of breast cancer in the Royal Marsden Hospital tamoxifen randomised chemoprevention trial. *Lancet* **352**: 98–101
Reed MJ (1995) The discriminant-function test: a marker of Th1/Th2 cell cytokine secretion and breast tumour oestrogen synthesis. *Mol Med Today* **1**: 98–103
Salber EJ, Trichopoulos D and MacMahon B (1969) Lactation and reproductive histories of breast cancer patients in Boston, 1965–66. *J Natl Cancer Inst* **43**: 1013–1024
Secreto G and Zumoff B (1994) Abnormal production of androgens in women with breast cancer. *Anticancer Res* **14**: 2113–2118
Secreto G, Recchione C, Cavalleri A, Miraglia M and Dati V (1993) Circulating levels of testosterone, 17-beta-oestradiol, luteinising hormone and prolactin in postmenopausal breast cancer patients. *Br J Cancer* **47**: 269–275
Secreto G, Toniolo P, Berrino F, Recchione C, Cavalleri A, Pisani P, Totis A, Fariselli G and Di Pietro S (1991) Serum and urinary androgens and risk of breast cancer in postmenopausal women. *Cancer Res* **51**: 2572–2576
Seymour-Munn K and Adams JB (1983) Estrogenic effects of 5-androsterone-3β-17β-diol and its possible implication in the etiology of breast cancer. *Endocrinology* **112**: 486–491
Thomas BS and Bulbrook RD (1966) Routine assay of urinary dehydroepiandrosterone, androsterone, and aetiocholanolone in urine by gas-liquid chromatography. In: Vermeulen A (ed.), *Androgens in Normal and Pathological Conditions*, pp. 49–53. Excerpta Medica Foundation: Amsterdam
Thomas BS and Walton DRM (1968) Chloromethyldimethylsilyl ethers in the routine assay of urinary 11-deoxy-17-oxosteroids by gas-liquid chromatography. *J Endocr* **41**: 203–211
Thomas HV, Key TJ, Allen DS, Moore JW, Dowsett M, Fentiman IS and Wang DY (1997*a*) A prospective study of endogenous serum hormone concentrations and breast cancer risk in premenopausal women on the island of Guernsey. *Br J Cancer* **75**: 1075–1079
Thomas HV, Key TJ, Allen DS, Moore JW, Dowsett M, Fentiman IS and Wang DY (1997*b*) A prospective study of endogenous serum hormone concentrations and breast cancer risk in postmenopausal women on the island of Guernsey. *Br J Cancer* **76**: 401–405
Thomas HV, Reeves GK and Key TJ (1997*c*) Endogenous estrogen and postmenopausal breast cancer: a quantitative review. *Cancer Causes Control* **8**: 922–928
Toniolo PG, Levitz M, Zeleniuch-Jacquotte A, Banerjee S, Koenig KL, Shore RE, Strax P and Pasternack BS (1995) A prospective study of endogenous estrogens and breast cancer in postmenopausal women. *J Natl Cancer Inst* **87**: 190–197
van Doorn LG, Poortman J, Thijssen JHH and Schwarz F (1981) Action and interactions of 5-androstene-3β,17β-diol and estradiol-17β in the immature rat uterus. *Endocrinology* **108**: 1587–1593
Veronesi U, Maisonneuve P, Costa A, Sacchini V, Maltoni C, Robertson C, Rotmensz N and Boyle P (1998) Prevention of breast cancer with tamoxifen: preliminary findings from the Italian randomised trial among hysterectomised women. *Lancet* **352**: 93–97
Wang DY, Moore JW, Thomas BS, Bulbrook RD, Hayward JL, Abe O, Utsunomiye J, Kumaoka S, Greenwood FC, Glober G and Stemmerman G (1979) Plasma and urinary androgens in women with varying degrees of risk of breast cancer. *Eur J Cancer* **15**: 1269–1274
Wang DY, De Stavola BL, Bulbrook RD, Allen DS, Kwa HG, Fentiman IS, Hayward JL and Millis RR (1992) Relationship of blood prolactin levels and the risk of subsequent breast cancer. *Int J Epidemiol* **21**: 214–221
Wang DY, De Stavola BL, Allen DS, Fentiman IS, Hayward JL and Bulbrook RD (1997) Breast cancer risk is positively associated with height. *Breast Cancer Res Treat* **43**: 123–128
Zeleniuch-Jacquotte A, Bruning PF, Bonfrer JMG, Koenig KL, Shore RE and Kim MY (1997) Relation of serum levels of testosterone and dehydroepiandrosterone sulfate to risk of breast cancer in postmenopausal women. *Am J Epidemiol* **145**: 1030–1038
Zhang Y, Kiel DP, Kreger BF, Cupples LA, Ellison RC, Dorgan JF, Schatzkin A, Levy D and Felson DT (1997) Bone mass and the risk of breast cancer among postmenopausal women. *N Engl J Med* **336**: 611–617

 *© 2000 Cancer Research Campaign*
